# Space-number association in zebrafish

**DOI:** 10.1016/j.isci.2026.118179

**Published:** 2026-11-09

**Authors:** Davide Potrich, Mirko Zanon, Rosa Rugani, Valeria Anna Sovrano, Giorgio Vallortigara

**Affiliations:** 1 Center for Mind/Brain Sciences, University of Trento, Rovereto, Italy; 2 Department of Psychology and Cognitive Science, University of Trento, Rovereto, Italy; 3 Department of General Psychology, University of Padua, Padova, Italy

**Keywords:** spatial-numerical association, mental number line, numerical cognition, number sense, behavior, zebrafish

## Abstract

Spatial-numerical associations reflect the tendency to map small numerosities to the left and larger numerosities to the right. Although widely documented in humans and some non-human species, its presence in teleost fish has remained unclear. Here, we investigated whether zebrafish exhibit a spatial-numerical association by presenting fish with a left-right numerical discrimination task in a controlled behavioral assay. Zebrafish exhibited systematic magnitude-dependent spatial choices, showing a significant spatial shift toward the left for smaller relative to larger numerosities across experimental conditions. These results provide evidence for a number-space association in zebrafish and suggest that number-space mapping may extend to basal vertebrate lineages. This highlights zebrafish as a promising model for investigating the neurobiological mechanisms underlying number-space associations.

## Highlights

• Zebrafish display relative spatial shifts according to numerical magnitude
• Spatial choices differ significantly between smaller and larger numerosity conditions
• Physical controls disproportionately affect the strength of spatial mapping for large sets

## Graphical abstract

**Figure:**
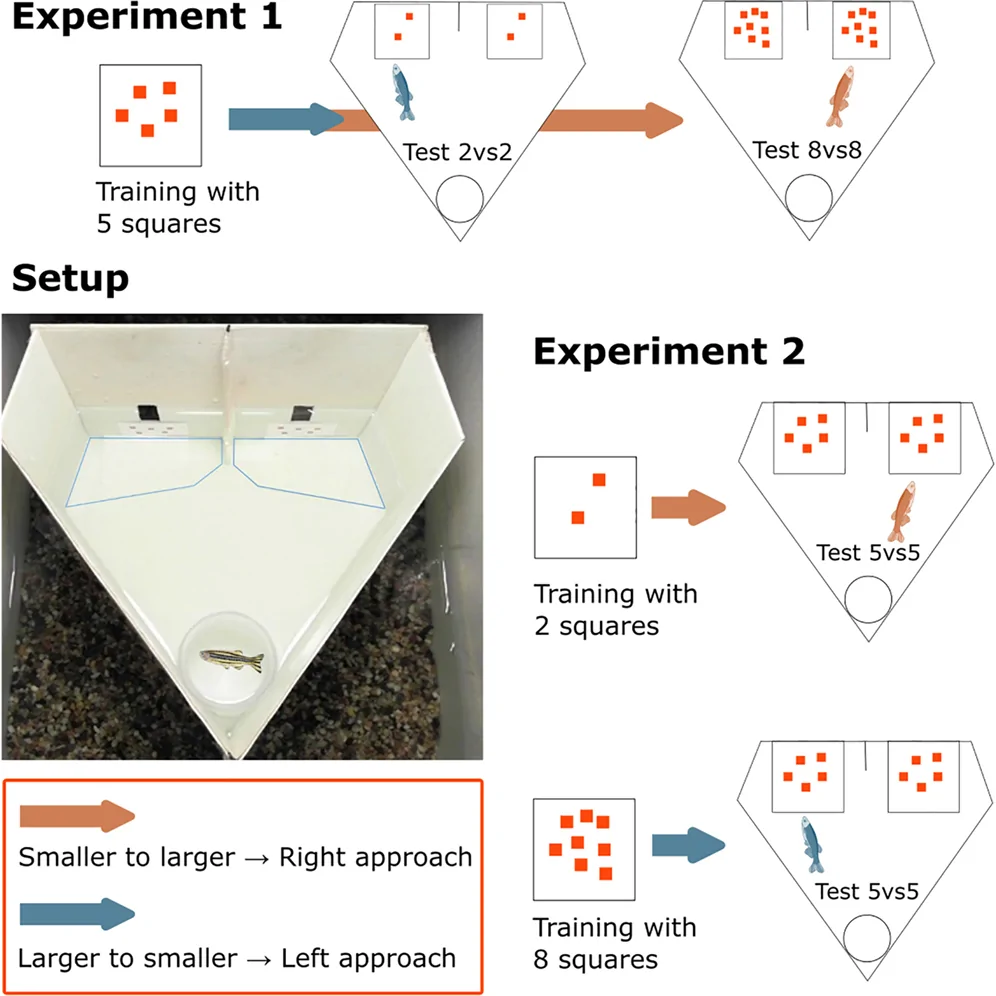

## Teaser

Piscine behavior; Biological sciences; Zoology

## Introduction

Spatial-numerical associations (SNA) describe a consistent behavioral tendency to map numerical magnitude onto space. In humans, smaller numbers bias responses leftward and larger numbers rightward, a pattern commonly interpreted as evidence for a spatially organized mental number line. Although first identified in adults (starting from Galton’s original observations^1^ and later formalized by Dehaene^2^ as the SNARC (Spatial-Numerical Association of Response Codes) effect), similar directional biases have since been documented across early development,^3^ and in populations with minimal formal education,^4^ indicating that space-number mapping does not depend on symbolic mathematics or cultural conventions.^5^

Importantly, numerical spatialization is not uniquely human. Converging evidence from primates, birds, and even invertebrates shows that non-human species associate numerical magnitude with space.^6^^,^^7^^,^^8^ Leftward biases for smaller numerosities and rightward biases for larger ones have been reported in domestic chicks and Clark’s nutcrackers,^9^^,^^10^ rhesus macaques,^11^^,^^12^ chimpanzees,^13^ and domestic dogs^14^; even honeybees display comparable space-magnitude mapping, despite their radically different neural architecture.^15^^,^^16^

However, the universality of this mapping across all numerical ranges remains debated. Although the overall pattern of small-left and large-right associations appears broadly conserved in birds and mammals, individual variability in directional preference has been documented in primates and birds,^17^^,^^18^^,^^19^ and contrasting evidence exists in other contexts.^20^^,^^21^

Two studies, respectively with domestic chicks and cleaner fish, provided a paradigmatic example.^10^^,^^21^ The seminal study on relative SNA in animals was conducted on domestic chicks, an animal model known to master a wide range of numerical abilities. During training, chicks learned to circumvent a panel displaying a target numerosity (5). At test, they faced two panels, one on the left and one on the right, depicting the same numerosity, either smaller (2) or larger (8) than the target. The animals showed a robust leftward bias when presented with the smaller numerosity and a rightward bias when presented with the larger one.^10^ This suggests that the numerosity experienced during training functions as a reference magnitude stored in memory, against which future numerosities may be compared. This aligns with evidence that zebrafish not only learn and remember visual information^22^^,^^23^ but also exhibit representational abilities, such as maintaining a goal-object in memory after limited exposure.^24^^,^^25^ Crucially, numerical cognition is thought to rely on two distinct non-symbolic systems: the approximate number system (ANS), which represents large magnitudes noisily and is ratio-dependent, and the object tracking system (OTS), which allows for the precise tracking of small sets (typically ≤4 items, also referred to as *subitizing* range).^26^^,^^27^ Notably, the capacity limit separating “small” from “large” numerosities is not absolute, but varies as a function of species, age, and task complexity.^26^^,^^28^^,^^29^ In teleost fish, the picture seems particularly complex, often revealing dissociations between these systems. For instance, mosquitofish (*Gambusia holbrooki, G. holbrooki*) show precise discrimination for small sets (1 vs. 2 up to 3 vs. 4) but fail at the 4 vs. 5 transition, requiring a larger ratio (1:2, e.g., 4 vs. 8) to discriminate larger magnitudes.^30^ Similarly, guppies (*Poecilia reticulata*) successfully compare quantities within the small (3 vs. 4) or large (5 vs. 10) ranges but fail specifically when comparisons closely span the system boundary (3 vs. 5), recovering performance only when the numerical distance is increased (e.g., 3 vs. 6).^31^ In contrast, other species such as angelfish (*Pterophyllum scalare*) appear to follow a more continuous ratio-dependent limit (Weber’s law) without a sharp discontinuity for small numbers.^32^ In zebrafish (*Danio rerio*), quantity discrimination is well-established across both small and large ranges,^33^ supported by specific neurons in the dorso-central pallium that respond to numerical change.^34^^,^^35^ Indeed, by comparing these previous findings in parallel with the diversity of experimental paradigms used, it acts as a compelling suggestion that the specific involvement of the OTS versus the ANS is not rigid, but may crucially depend on task-specific demands and the cognitive load imposed by the experimental setting. This heterogeneity suggests that spatial-numerical mapping in fish may not be a generalized trait, but rather one that depends on the specific cognitive system (exact vs. approximate) recruited.

The two studies mentioned earlier, respectively with domestic chicks and cleaner fish, provided a paradigmatic example.^10^^,^^21^ Chicks were trained to circumvent a panel displaying a target numerosity (5) and subsequently tested with panels depicting either a smaller (2) or larger (8) numerosity. This design explicitly crosses the boundary between the small number range (subitizing/OTS) and the large number range (ANS). The animals showed a robust leftward bias when presented with the smaller numerosity and a rightward bias when presented with the larger one.^10^ Yet, when a similar experimental paradigm was applied to cleaner fish (*Labroides dimidiatus*, *L. dimidiatus*), which succeeded in a numerical discrimination task, no systematic numerical spatialization was observed.^21^ This failure might depend on the numerosities used, even though fish succeeded in a numerical discrimination task requiring them to distinguish between two simultaneously visible quantities. Another possible explanation can be temporal stability of numerical information in working memory, as supported by evidence that cleaner wrasse fails in both visual and spatial working memory tasks,^36^ indicating a bottleneck in maintaining representations over time. By contrast, the paradigm was originally designed for chicks, taking into account their working-memory duration and the specific experimental constraints,^10^ and using magnitudes that chicks had already been shown to discriminate successfully.^27^^,^^28^

Two main evolutionary scenarios remain plausible. The presence of SNA in taxa that diverged more than 500 million years ago—such as honeybees and vertebrates—suggests that this mapping between number and space may represent an ancient, conserved cognitive mechanism. Alternatively, SNA may have arisen independently in different lineages through convergent evolution, as an adaptive response to shared ecological demands. The heterogeneous findings in fish further complicate this picture: if the absence of SNA in some aquatic species is confirmed, it may indicate either a later emergence of the trait within specific vertebrate clades or a secondary loss in certain environments. Determining whether SNA reflects a deep ancestral heritage or a convergent cognitive adaptation remains an open challenge for comparative research.^37^ At the cognitive level, these findings suggest that SNA may arise from evolutionarily ancient mechanisms, but its expression may differ based on the cognitive architecture used to process numerosities, such as the capacity and duration of working memory required to bridge the stimulus-to-response interval. The robustness of the chick paradigm, which has been successfully applied in other species,^3^^,^^15^^,^^38^ combined with the informative null result in cleaner fish underscores the need for systematic investigations in model organisms like zebrafish to disentangle these factors. Zebrafish have already demonstrated the capacity to use numerical information in ordinal counting tasks^39^ and proto-arithmetic contexts,^40^ while showing efficient one-trial memory processing.^41^

In this study, we investigated whether zebrafish exhibit an SNA bias and whether this bias holds consistent when crossing the cognitive boundary between small and large numbers. We adapted a controlled behavioral task in which fish were required to discriminate between numerosity cues presented laterally (see Figure 1). Specifically, fish were initially trained to discriminate between a stimulus depicting a fixed numerosity (experiment 1: five squares; experiment 2: two or eight squares) and a blank stimulus. The choice of these specific numerosities (2, 5, and 8) allowed us to test the subjects’ responses to magnitudes that fall within the distinct cognitive ranges typically associated with the OTS and the ANS, while keeping a framework directly comparable with previous works.^10^^,^^21^

**Figure 1:**
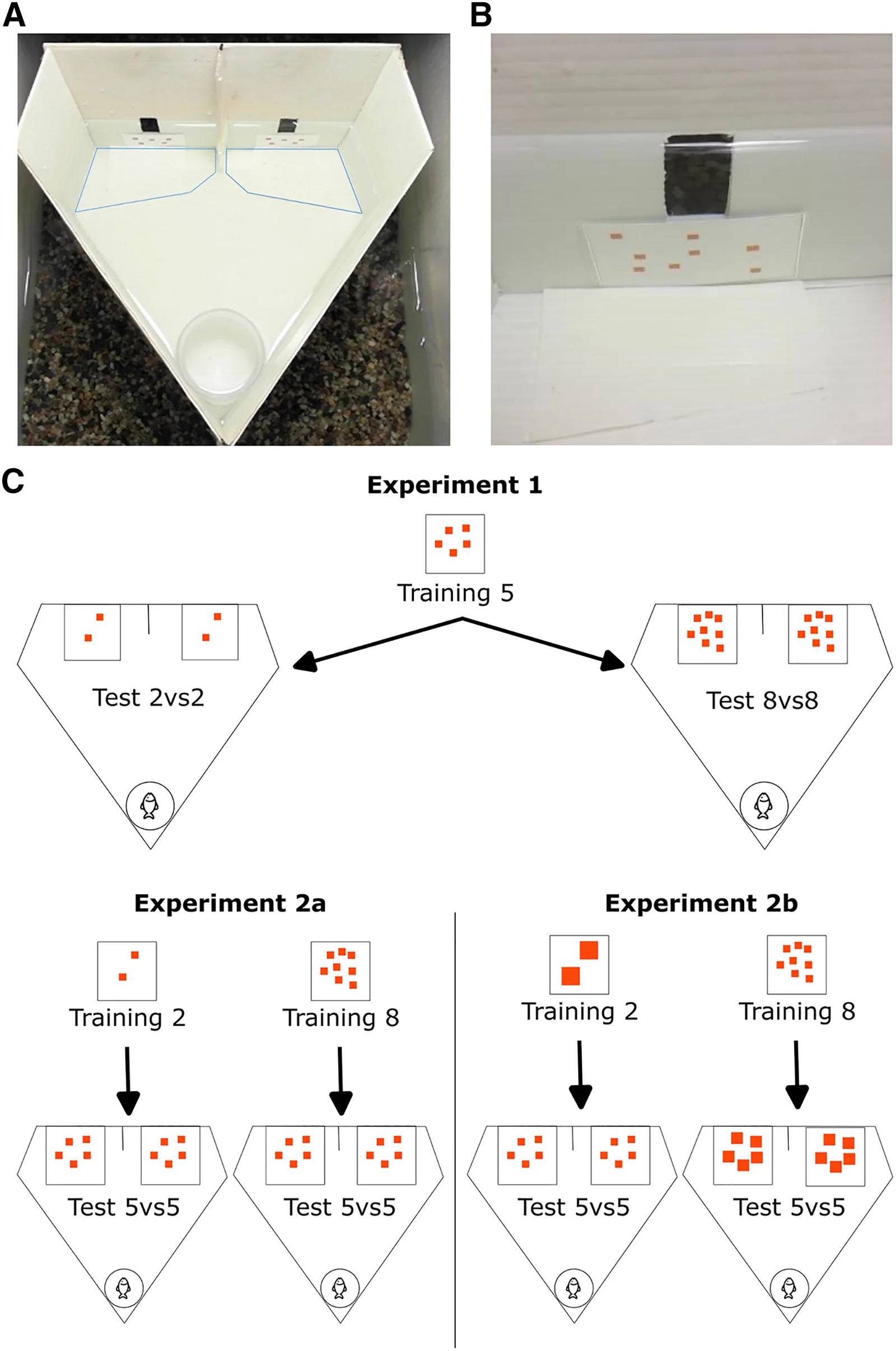
Apparatus and experiments schematic (A) Image of the apparatus. Decision areas are highlighted in blue. (B) Image of the apparatus exit door with an example of the numerosity stimulus. (C) Schematic of the two main experiments: experiment 1 consists of a training with 5 squares followed by two tests (2 vs. 2 squares and 8 vs. 8), while experiment 2 consisted of two groups, one trained with 2 squares and tested with 5 vs. 5, and one trained with 8 squares and tested with 5 vs. 5. In experiments 1 and 2a, all squares have the same dimensions, while in experiment 2b, the squares’ overall perimeter is preserved between training and test stimuli.

If fish associate numerosity with space across these ranges, in experiment 1, they would select the left stimulus in the 2 vs. 2 test and the right one in the 8 vs. 8 test. If their SNA is relative, in experiment 2a, facing the same 5 vs. 5 test, we expect them to approach the left stimulus after training with 8 and the right one after training with 2. Finally, experiment 2b controlled for key physical variables (perimeter, which is also related to area), although not all continuous variables were manipulated, to assess whether performance could be explained by some of the most well-established non-numerical perceptual cues.

A second hypothesis is that SNA in fish may be restricted to the small-number range or to large numerosities within large discernible numerical ratios. Under this account, our design should still be able to detect it: in experiment 1, fish would be expected to choose the left stimulus in the 2 vs. 2 test after training with 5. The design also allows us to test the relativity of SNA: in experiment 2, following training with 2, fish should preferentially select the right stimulus in the 5 vs. 5 comparison. Experiment 2b further evaluates whether such choices persist when continuous physical variables (like perimeter and area) are controlled for. A delimitation to the small-number range would be supported by chance-level performance whenever 8—falling within the large-number range—is used.

## Results

Fish successfully reached the learning criterion after an average of 99 ± 16 trials for experiment 1, 172 ± 21 trials for experiment 2, and 84 ± 10 trials for experiment 2b. We did not detect any correlation between the number of trials required to reach learning criterion and task performance across all experiments (Spearman’s *ρ* = 0.07, *p* = 0.643; see Figure S1).

Test results for the different experiments are reported in Figure 2.

**Figure 2:**
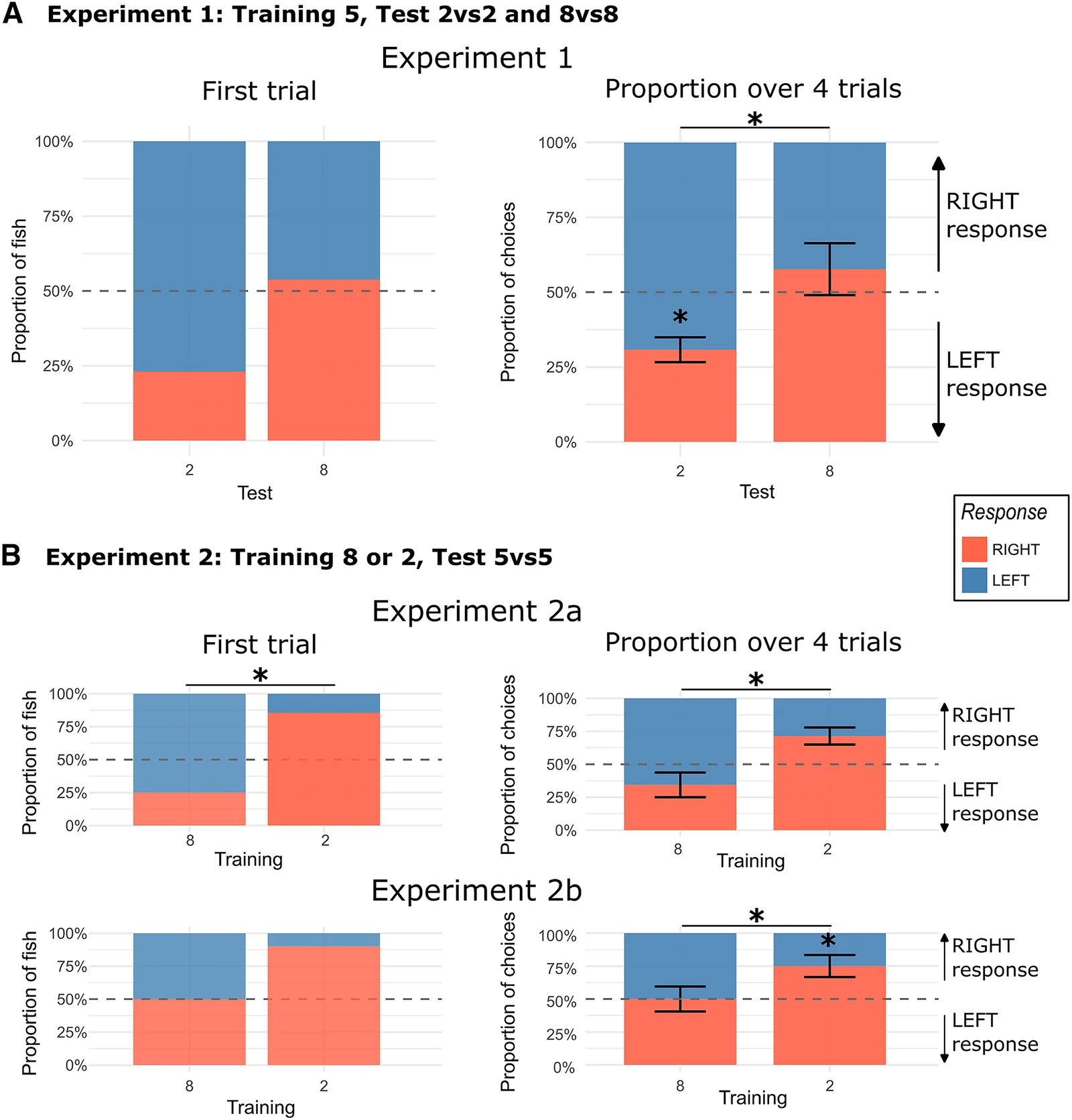
Experimental test results. Proportion of choices on the first test trial (left, proportion of fish) and across all four test trials (right, proportion of choices) for the different experimental conditions (A) Experiment 1: fish were trained with 5 squares and subsequently tested with 2 vs. 2 and 8 vs. 8 squares in a within-subjects design. (B) Experiment 2: fish were trained with either 2 or 8 squares and tested with 5 vs. 5 squares in a between-subjects design. The upper plots show experiment 2a, in which stimulus perimeter covaried with numerosity, whereas the lower plots show experiment 2b, in which overall stimulus perimeter was controlled. Dashed horizontal lines indicate chance level (50%). Asterisks ∗ indicate *p* values < 0.05 (Holm-adjusted comparisons against chance level and between-condition comparisons, as appropriate; see STAR Methods for details).

### Different spatial responses to smaller versus larger numerosities

In experiment 1, fish were trained on 5 squares and then presented with either a smaller (2 vs. 2) or larger (8 vs. 8) numerosity in separate test sessions.

Critically, a mixed-effects logistic regression evaluating all test trials revealed a significant main effect of test condition, indicating that fish exhibited different spatial responses when presented with smaller versus larger numerosities (type 3 Wald *χ*^2^ = 7.43, *p* = 0.006; odds ratio [test 2/test 8] = 0.326, 95% confidence interval [CI, 0.146, 0.730]).

Post hoc analyses of the estimated marginal means against chance (50%) confirmed that when presented with 2 vs. 2 squares, fish showed a significant leftward bias across all trials (probability of choosing right = 0.308, 95% CI [0.198, 0.445], Cohen’s h = −0.394, Holm-adjusted *p* = 0.014). Conversely, when presented with 8 vs. 8 squares, fish did not show a significant rightward bias (probability of choosing right = 0.577, 95% CI [0.440, 0.703], Cohen’s h = 0.155, Holm-adjusted *p* = 0.269), although the direction of the response remained consistent with SNA predictions.

When restricting the analysis to the first trial only, the direct within-subjects comparison between test conditions was not statistically significant (exact McNemar’s test, *p* = 0.125, Cohen’s g = 0.50).

### Stronger spatial mapping following training with small numerosities

In experiment 2, separate groups of fish were trained on either 2 or 8 squares and then presented with 5 vs. 5 squares at test.

Critically, a mixed-effects logistic regression evaluating all test trials revealed a significant main effect of training condition, demonstrating that fish trained with different numerosities exhibited significantly different spatial responses when experiencing the same intermediate test numerosity (type 3 Wald *χ*^2^ = 7.79, *p* = 0.005; odds ratio [training 2/training 8] = 4.773, 95% CI [1.593, 14.302]).

Post hoc analyses of the estimated marginal means against chance (50%) revealed that fish trained with 2 squares (when presented with a larger numerosity at test) showed a marginally significant trend toward a rightward bias across all test trials with 5 squares (probability of choosing right = 0.714, 95% CI [0.524, 0.850], Cohen’s h = 0.442, Holm-adjusted *p* = 0.057). Fish trained with 8 squares (when presented with a smaller numerosity at test) similarly showed a non-significant leftward trend across all trials (probability of choosing right = 0.344, 95% CI [0.202, 0.521], Cohen’s h = −0.317, Holm-adjusted *p* = 0.082), though choices in both groups were directionally consistent with SNA expectations.

When restricting the analysis to the first trial only, the direct between-subjects comparison remained significant, indicating a strong divergence in initial choices between the two training conditions (likelihood ratio *χ*^2^ = 5.99, *p* = 0.014; odds ratio = 18.000, 95% CI [1.267, 255.743]). However, when evaluating these first-trial choices against chance, neither the 2-square training group (probability of choosing right = 0.857, Cohen’s h = 0.795, Holm-adjusted *p* = 0.194) nor the 8-square training group (probability of choosing right = 0.250, Cohen’s h = −0.524, Holm-adjusted *p* = 0.194) reached statistical significance after adjusting for multiple comparisons.

### Asymmetric robustness of small versus large magnitude mapping to continuous variable controls

In experiment 2b (experiment controlling for continuous variables, i.e., perimeter), a mixed-effects logistic regression evaluating all test trials revealed a significant main effect of training condition, demonstrating that fish trained with different numerosities exhibited significantly different spatial responses when experiencing the same intermediate test numerosity (type 3 Wald *χ*^2^ = 3.93, *p* = 0.048; odds ratio [training 2/training 8] = 3.182, 95% CI [1.012, 9.999]).

Post hoc analyses of the estimated marginal means against chance (50%) confirmed that fish trained on 2 squares maintained a significant SNA-consistent rightward bias when presented with 5 squares across all test trials (probability of choosing right = 0.761, 95% CI [0.580, 0.880], Cohen’s h = 0.549, Holm-adjusted *p* = 0.013). In contrast, fish trained on 8 squares exhibited no lateral bias, with performance dropping to chance levels (probability of choosing right = 0.500, 95% CI [0.313, 0.687]), indicating an absence of numerical spatialization in this condition.

When restricting the analysis to the first trial only, the direct between-subjects comparison showed a marginally significant trend toward different spatial responses (likelihood ratio *χ*^2^ = 3.68, *p* = 0.055; odds ratio = 9.000, 95% CI [0.748, 108.295]). Evaluating these first-trial choices against chance revealed a similar pattern: a marginally significant rightward trend for the 2-square training group (probability of choosing right = 0.900, Cohen’s h = 0.927, Holm-adjusted *p* = 0.074) and no bias for the 8-square training group.

## Discussion

The present study provides evidence that zebrafish spatial choices are systematically influenced by numerical magnitude, showing a pattern consistent with a SNA and with spatial-numerical mappings described in humans and other vertebrates.^6^^,^^37^ Fish trained on a reference numerosity and then presented with smaller or larger numerosities showed systematic shifts in spatial preference depending on the numerical relationship between training and test stimuli. While these results support a numerical contribution to spatial choices, because multi-dimensional continuous controls (such as element density and convex hull) were not applied in experiment 2b, we cannot fully rule out the combined influence of continuous visual magnitudes alongside numerical cues. Nonetheless, these findings suggest that spatial-numerical associations may extend beyond mammals and birds, supporting the possibility that links between numerical magnitude and space are widespread across vertebrates. Moreover, this extends the study of space-number mapping to a genetically tractable vertebrate model, providing a promising framework for investigating the neural and evolutionary foundations of numerical cognition.

Our results reveal intriguing asymmetries in the strength of SNA depending on training numerosity, which may reflect distinct processing mechanisms for small versus large magnitudes. Fish trained with small numerosity (2 or 5) consistently exhibited robust spatial biases across test conditions. However, associations involving the larger numerosity (8) were less reliable: training with 5 and testing with 8 produced directionally consistent but non-significant biases, while training with 8 and testing with 5 yielded a robust between-group difference in spatial bias (*p* = 0.005). This pattern is compatible with recent evidence highlighting a role for absolute magnitude in spatial-numerical associations. As noted by Roth et al.,^42^ lower numerical ranges tend to produce stronger SNA, where both relative and absolute small numbers are associated with left space, while only relatively large numbers are linked to right space. As a hypothesis for future research, this asymmetry could potentially reflect differences in numerical precision across ranges (e.g., precise representations characteristic of an OTS vs. ratio-dependent noise of an ANS). However, because our paradigm evaluated a restricted set of numerosities,(2, 5, and 8) whether distinct core number systems (OTS vs. ANS) drive these spatial choices remains an open hypothesis that requires systematic empirical testing across broader magnitude ranges. In line with this possibility, the 5 vs. 8 contrast (ratio 0.625) sits immediately adjacent to the known limit of zebrafish numerical precision. Previous work demonstrates that while zebrafish can discriminate at a 0.67 ratio (4 vs. 6), they fail at 0.75 (6 vs. 8) and also struggle with the 3 vs. 4 small-number limit.^33^ This suggests that the 5 vs. 8 comparison is inherently demanding; thus the reduced reliability of numerical representations at this range may contribute to weaker spatial biases compared with those observed for smaller numerosities. This destabilization may be further exacerbated by limits in visual memory. While zebrafish exhibit efficient one-trial memory in simpler contexts,^41^ the cognitive load of maintaining a representation of “8” might lead to a comparison drawback during the decision-making process.

A related hypothesis to be evaluated in future work is whether small- and large-number ranges show differential resilience to continuous stimulus variations. Fish trained on 2 squares maintained robust SNA biases when presented with 5 squares at test, even when perimeter cues were controlled. In stark contrast, fish trained on 8 squares showed no lateral bias, with performance dropping to chance levels. Critically, a direct between-group comparison revealed that fish trained with different numerosities exhibited significantly different spatial responses (*p* = 0.048), even when perimeter cues were controlled. This asymmetry suggests that fish trained with larger numerosity may rely more strongly on continuous magnitude cues during learning, and when these cues are dissociated from numerosity at test, the resulting conflict disrupts spatial mapping. Under a dual-system framework, one potential hypothesis is that the OTS may be more resilient to continuous physical variations like perimeter variations, focusing instead on discrete item identity.^26^^,^^27^^,^^34^ However, because other non-numerical variables such as element density and convex hull were not controlled, and given that only three numerosities were tested, whether this performance asymmetry reflects the involvement of an OTS remains a speculative hypothesis for future empirical investigation.

Importantly, the observed pattern is unlikely to be explained solely by a simple fixed individual- or population-level lateralization, which would typically manifest as a consistent side choice regardless of the numerical condition (see e.g., in chicks^43^). Because fish systematically shifted their spatial preference based on whether the stimulus was smaller or larger than the reference, this dynamic choice behavior makes a fixed baseline side bias less likely. Nevertheless, context-dependent lateralization elicited by specific stimulus or cognitive demands cannot be entirely excluded, though the overall pattern provides support for magnitude-dependent spatial choices in zebrafish.

When compared with existing evidence, our results highlight a contrast between the present study and the findings reported by Triki and Bshary^21^ in the cleaner fish *L. dimidiatus*. Specifically, *L. dimidiatus* showed no evidence of a spatial-numerical association, despite using similar numerosities. This discrepancy in SNAs across teleost species could reflect niche-specific adaptations. While the underlying neurobiological mechanisms may be conserved, their behavioral manifestation depends heavily on the ecological relevance of the task and the evolutionary pressures shaping a species’ navigation. For instance, species-specific spatial strategies and varying capacities for working or reference memory may exist,^23^ even when other paradigms (such as detour experiments^25^) fail to differentiate between marine and freshwater populations. This highlights that the choice of experimental paradigm could be critical in revealing possible cognitive divergences. In Triki and Bshary’s procedure,^21^ cleaner fish had to swim around a central stimulus, a design that may have allowed subjects to solve the task without actively attending to numerosity. In our paradigm, by contrast, attentional engagement with the numerical stimulus was deliberately emphasized, as reinforcement was contingent solely on selecting the panel with a specific numerosity of elements. This aspect is especially relevant for zebrafish, which require explicit task relevance to direct attention to specific stimulus features.^44^ Future comparative work should clarify whether the system for small numbers (as seen in *G. holbrooki*) is universally linked to spatial mapping, or if species-specific ecological constraints decouple these systems. A more fundamental explanation for this discrepancy may lie in inherent differences in working memory capacity. Recent comparative studies indicate that cleaner fish (*L. dimidiatus*) perform poorly in both visual and spatial working memory paradigms,^36^ potentially explaining their failure to exhibit SNA. In contrast, zebrafish possess a more robust memory architecture, enabling them to integrate numerical and spatial information.^39^ Working memory in zebrafish is also confirmed by their ability to handle numerical information in proto-arithmetic contexts,^40^ and their efficient one-trial memory performance.^41^ This raises the possibility that a minimum threshold of working memory stability is a prerequisite for the emergence of SNA.

In conclusion, the present findings highlight zebrafish as a promising model for investigating the biological mechanisms underlying spatial-numerical associations and motivate future research. The observation of SNA-like spatial biases in zebrafish is particularly significant given the species’ status as a leading genetic model organism.^45^ The extensive genetic toolkit available for zebrafish, including CRISPR-Cas9 gene editing, transgenic lines with fluorescently labeled neurons, and well-characterized neural circuits, positions this species as an ideal system for dissecting the biological foundations of numerical cognition.^35^^,^^46^^,^^47^^,^^48^ Developmental studies could determine whether these mappings are innate or shaped by specific experiences. Genetic approaches could help identify candidate genes and neural pathways via targeted knockouts or mutant screening. Calcium imaging could localize brain regions mediating these effects, potentially revealing whether conserved neural mechanisms contribute to spatial-numerical mapping across vertebrates. Future work could also test broader ranges of numerosity and systematically manipulate continuous variables to determine their contributions to spatial choices. Additionally, studies could examine individual differences in spatial biases and investigate whether training can modulate spontaneous SNAs.

By bridging comparative psychology and molecular neuroscience, zebrafish research can provide unique insights into how abstract numerical information is represented and spatially organized in the mind. Together with evidence across other taxa (including humans, non-human primates, birds, and now fish), the present findings offer an initial demonstration of magnitude-dependent spatial choices in zebrafish, providing a valuable foundation for testing whether space-magnitude associations reflect a shared ancestral trait or convergent cognitive adaptations across vertebrates. Zebrafish, with their unique combination of demonstrable numerical abilities and powerful genetic and neurobiological tools, provide an unprecedented opportunity to investigate the mechanisms underlying this phenomenon, potentially shedding light on the evolution and development of numerical cognition across species.

### Limitations of the study

While our results provide the first evidence of numerical magnitude-related spatial biases consistent with SNA in zebrafish, several limitations must be considered.

First, the number of independent experimental units (fish) in each experiment was relatively small, which may have reduced statistical power and limited our ability to detect individual differences. To make the most efficient use of the available data, we analyzed the repeated binary choices using a unified generalized linear mixed model (GLMM) framework, which accounts for within-subject variation while appropriately treating fish identity as a random effect. Although this approach increases statistical efficiency and confirms robust effect sizes (odds ratios) for the small-numerosity conditions, it does not overcome the limitation imposed by the modest sample size. The first-trial analysis provides a more stringent assessment of the initial response and, although not all comparisons reach statistical significance, showed a direction of effect consistent with the four-trial analysis, supporting the overall pattern while indicating that the evidence is stronger when responses across the repeated test trials are considered. Future studies with larger cohorts will also be better positioned to characterize potential individual differences in SNAs^17^^,^^18^^,^^19^ (see individual scores in Figure S2).

Regarding continuous physical variables, our control condition (experiment 2b) was limited to equating the total perimeter of the stimuli. This manipulation effectively made the total surface area inversely proportional to numerosity, thus putting area cues in contrast with numerical cues. However, we did not systematically control for other non-numerical variables such as density or convex hull. While the robustness of the “small number” results suggests that zebrafish can process discrete item identity despite these variations, the disruption of performance in the “large number” condition suggests that these continuous magnitudes may play a stronger role when the ANS is recruited. Future studies should employ multi-variable controls to disentangle the specific contributions of density, area, and convex hull across a wider range of numerosities.

The current study tested a limited set of numerosities.(2, 5, and 8). To determine whether the observed asymmetry reflects differences between the OTS and ANS in zebrafish, future work should employ a broader range of values (e.g., involving numerosities 3, 4, and also bigger than 8) to precisely identify the point at which the spatial mapping degrades or shifts strategy. Moreover, the transition from training to test imposes a specific cognitive load. In experiment 2 (the 5 vs 5 test), the fish must retrieve a reference value from memory and compare it to the current stimulus. If this comparison exceeds the species’ processing limit (as suggested by the lack of success in other teleosts with poor working memory^36^) the spatial-numerical mapping may fail.

Finally, we did not measure baseline lateralization before training. Brain lateralization has been shown to modulate SNAs in other vertebrates^49^ and lateralization in fish has been reported to vary across behavioral contexts^50^; future studies could include an independent assessment of baseline lateralization before training to further exclude any contribution of context-dependent lateralization. While the observed reversal of spatial choices across conditions renders a simple fixed side bias less likely, future studies should incorporate independent pre-test assessments of lateralization across varied behavioral contexts to more fully dissociate numerical mapping from context-induced lateralized biases.

## Resource availability

### Lead contact

Further information and requests should be directed to and will be fulfilled by the corresponding author, Giorgio Vallortigara (giorgio.vallortigara@unitn.it).

### Materials availability

This study did not generate new unique reagents nor new animal lines.

### Data and code availability

• Data is available in Figshare repository: https://doi.org/10.6084/m9.figshare.30869576.
• Code used for the analysis of this work is available in Figshare repository: https://doi.org/10.6084/m9.figshare.30869576.
• Any additional information can be required to corresponding contact.

## Acknowledgments

This project was funded by the 10.13039/501100000781European Research Council under the European Union's Horizon 2020 research and innovation programme, grant agreement no. 833504 (SPANUMBRA) to G.V.; by FARE–Ricerca in Italia: Framework per l'Attrazione e il Rafforzamento delle Eccellenze per la ricerca in Italia, III edition, project “NUMBRISH–the neurobiology of numerical cognition: searching for a molecular genetic signature in the zebrafish brain” prot. R20YL9WN9N to G.V.; by PRIN (Progetti di Rilevante Interesse Nazionale, 2019) “number-space association: a comparative developmental and neurobiological approach” to G.V., V.A.S., and 2022 grant agreement no. P2022TKY7B (PNRR): “the emergence of proto-arithmetic abilities with empty and non-empty sets” to G.V. This project has also received support from the Italian Ministry of University and Research through the Research Project of National Relevance (PRIN), 2022 prot. 202254RHRT, to R.R. We wish to thank Nicola Corbellini, Chiara Franceschi, Francesca Morbioli, Anna Mutti, Alessia Panizza, Elisabetta Pedrocco, Valerio Rubino, Alessandra Venezia, and Johannes Waibl, for their partial assistance with the data collection; Ciro Petrone and Michela Maffei for the animal care and welfare.

## Authors contribution

Conceptualization, D.P., G.V., R.R., and V.A.S.; setup implementation, D.P.; data analysis, M.Z.; funding acquisition, G.V. and V.A.S.; supervision, G.V., V.A.S., D.P., and R.R.; writing original draft, D.P. and M.Z. All authors reviewed and edited the final version of the manuscript.

## Declaration of interests

The authors declare no competing interests.

## STAR★Methods

### Key resources table

**Table:** 

REAGENT or RESOURCE	SOURCE	IDENTIFIER
**Experimental models: Organisms/strains**		

Zebrafish (*Danio rerio*)	Local commercial hatchery (“Acquario G″ di Segatta Stefano, Trento, Italy)	NCBI TaxID: 7955

**Deposited data**		

Data for behavioral choices	This paper	https://doi.org/10.6084/m9.figshare.30869576
R script for statistical data analysis	This paper	https://doi.org/10.6084/m9.figshare.30869576

**Software and algorithms**		

RStudio version 2025.09.2 + 418	Posit Software, PBC	https://posit.co/download/rstudio-desktop/
G∗Power version 3.1.9.6	HHU	https://www.psychologie.hhu.de/arbeitsgruppen/allgemeine-psychologie-und-arbeitspsychologie/gpower

**Other**		

Submergible internal filter (NEWA Micro IF, MCF 40)	NEWA Tecno Industria	Cat# 00.24.055; EAN: 8017003074008
ZebTEC Benchtop Standalone Housing System	Tecniplast	Cat# ZB10BS; RRID: SCR_026364
Incandescent light bulb (60W)	Philips	Cat# 01150305; EAN: 8711500011503
Sera GVG-Mix flake food	Sera	Cat# 00254; EAN: 4001942002548
White polypropylene sheet (Poliplak)	Poliplak	CAS: 9003-07-0; SKU: Poliplak White
Microsoft LifeCam Studio HD Webcam	Microsoft	Cat# Q2F-00013 (od 5WH-00002); Model: 1425

### Experimental model and study participant details

In the experiments, adult male zebrafish (*Danio rerio*) served as test subjects, while female zebrafish were used as a social reward. These rewards were housed in an ‘open water’ area outside the choice tank and were not visible to the subjects during the decision phase. Consequently, the physical characteristics of the females could not influence the choice, as they served solely as a post-choice motivational anchor. Fish were provided by a local pet shop (“Acquario G” di Segatta Stefano, Trento, Italy) housed in 3.5 L plastic tanks within an automated aquarium system (ZebTEC Benchtop, Tecniplast), in same-sex groups of 10 individuals. Water temperature was maintained at 26 °C, with a 12:12 - h LD cycle. Animals were fed dry food (Sera GVG-Mix) three times per day.

During the experimental period, subjects were individually housed in 20-L plastic aquaria (23 × 38 × 25 cm) containing gravel and artificial vegetation to simulate a naturalistic environment. Although physically isolated, fish had visual access to conspecifics through a mesh divider, minimizing potential effects of social deprivation. Total body length ranged from 4 to 5 cm across subjects.

Fifteen fish were observed in Experiment 1, of which 13 reached the learning criterion and subsequently proceeded to the test phase. Experiment 2a involved 20 fish, of which 15 reached the criterion (7 trained with 2 elements and 8 trained with 8 elements). Experiment 2b involved 20 fish, of which 18 fish reached the criterion (10 trained with 2 elements and 8 trained with 8 elements). Fish in each experiment were experimentally naive, and therefore different subjects were engaged in the different experiments.

All experimental procedures complied with current Italian and European Union legislation regarding the ethical use of animals in research. The study was approved and licensed by the Italian Ministry of Health (auth. no. 1111/15-PR, prot. no. 13/2015 and auth. no. 893/2018-PR).

### Method details

#### Apparatus and stimuli

The experimental setup consisted of a diamond-shaped arena (28 × 25 × 19 cm) made of white plastic (Poliplak; see Figure 1A). Two identical rectangular exit openings (1.5 × 4.5 cm) were symmetrically positioned on the longest wall of the arena, 4.5 cm above the bottom. These openings allowed access from the central arena to an external compartment.

The arena was placed inside a larger opaque tank (35 × 49 × 27 cm) containing gravel, artificial plants, and two female conspecifics, which provided a strong social incentive for subjects to exit the arena. Directly below each exit opening, a removable white plastic panel displayed a fixed number of orange squares serving as the numerical stimuli (see Figure 1B). A vertical plastic divider protruded between the two exits, separating the arena into left and right choice zones.

Water temperature was kept at 26 °C and water was circulated using a pump and filtration system (Micro Jet Filter MCF 40). Illumination was provided by a 60 W incandescent lamp, and fish behavior was recorded from above using a webcam (Microsoft LifeCam Studio) placed approximately 50 cm above the apparatus.

The stimuli consisted of two-dimensional orange (RGB: 252,72,11) squares printed on laminated white cards. For each stimulus, the size of the individual squares was held constant, while their spatial arrangement varied randomly (with a minimum inter-distance of 4 mm and maximum of 21 mm). For each numerosity, eight distinct spatial configurations were created. In Experiments 1 and 2, all squares had a side length of 3 mm. These squared elements, viewed from a distance of 17 cm (starting position-stimuli distance), subtend a visual angle of approximately 1°. Considering a minimum inter-stimulus gap of 3 mm, the resulting spatial frequency is approximately 0.5 cycles per degree (cpd). Because the actual inter-distance values were typically larger than 3 mm, which would result in an even lower and more easily resolvable spatial frequency, this represents a conservative estimate. This value remains below the reported visual acuity threshold for adult zebrafish (approximately 0.6 cpd^51^), ensuring the stimuli were clearly resolvable already from the starting position. In Experiment 2b (experiment controlling for continuous variables), stimulus size ranged from 3 mm to 7.5 mm. In this latter condition, the continuous non-numerical variable controlled across different numerosity was the overall perimeter. Balancing the perimeter, allowed us to evaluate its role in the spatial association and to indirectly assess the influence of overall area. Specifically, when the perimeter was equalized between two arrays displaying different numerosity, the stimulus with fewer elements had a larger total area than the one with more elements.^52^ If the animal had relied on the overall area of the stimulus rather than the number, a reversed spatial numerical preference would have been observed.

#### Behavioural assay

The experiment consisted of a training phase followed by a test phase. During training, only one of the two exit doors was accessible, while the other was blocked using a transparent plastic sheet. The open door was indicated by a plastic card displaying a fixed number of elements, whereas a blank white card was positioned beneath the blocked door. Each fish underwent one daily session consisting of 10 trials. Before the start of each session, the subject was transferred from its home aquarium to the outer, enriched environment for a 5- min acclimation period. At the beginning of each trial, the fish was confined within a transparent plastic cylinder (6 cm diameter) positioned in front of the stimuli. After a 15-s delay, the cylinder was raised, allowing the fish to explore the arena freely. A correction procedure was applied: if an incorrect door was approached, the fish was allowed to revise its choice until it successfully exited through the open door or until the maximum trial duration of 15-min had elapsed. A choice was recorded when the fish contacted a door with its snout (in the case of the blocked door) or passed through the open door. Reinforcement was contingent on performance, such that a correct first choice resulted in a 6-min access to food and social companions, whereas one or more incorrect choices resulted in a 3-min interval without food and conspecifics. If no choice was made within the allotted time, the fish was given a 5-min pause. Across trials, the side of the reinforced exit (left or right) and the spatial configuration of the elements were randomized to ensure that numerosity was the only reliable cue. Learning was considered achieved when a subject reached at least 70% correct choices in two consecutive sessions, after which it proceeded to the test phase.

In the test phase, subjects completed four unrewarded trials with both exit doors closed. The test phase consisted of four trials. In each trial, two identical stimuli depicting numerosity different from that used during training were placed beneath the left and right exits. Restricted choice areas were delineated in front of each door using white plastic panels on the tank floor, defining discrete left and right zones and minimizing a central neutral region (see Figure 1A; areas marked in blue). Before each trial, the fish was again confined in the cylinder and released after 15-s; the first approach was recorded when the fish entered one of the zones with its entire body. Inter-trial intervals lasted 5-min, during which the fish could freely swim in the outer environment. For each subject, both the direction of the first approach and the total number of approaches across the four test trials were analyzed. The four-trial analysis was considered the primary measure of spatial preference because the test was designed to assess directional choice across repeated unrewarded trials: the within-subject analysis provides more information per subject, which is particularly relevant given the relatively small sample size, while reducing the influence of stochastic exploration on any single choice. The first-trial response was analyzed separately as a complementary measure of the initial spontaneous preference.

Two main experiments were conducted. Experiment 1 employed a within-subjects design in which fish were trained on a target numerosity of 5 squares and subsequently observed in tests with numerosity of 2 vs. 2 and 8 vs. 8 squares; the order of the two test conditions was counterbalanced across subjects. Experiment 2 used a between-subjects design in which fish were trained on either 2 or 8 elements and observed in the test with a numerosity of 5 vs. 5 squares. Experiment 2 was further divided into two variants: Experiment 2a, in which continuous quantitative properties covaried with numerosity, and Experiment 2b, in which stimuli were controlled for overall perimeter that remained constant across training and test stimuli.

All test trials were recorded and scored offline using a double-blind procedure (by two scorers), based on clear physical markings on the floor of the experimental tank: entry into a choice zone was marked as a binary event. Cohen’s kappa (κ) was used to evaluate the interobserver reliability based on 20% of the trials, which revealed a perfect interobserver agreement (κ = 1.0).

### Quantification and statistical analysis

All analyses were performed in R (R Core Team) within RStudio (version 2026.04.0 + 526). Data were analyzed using Generalized Linear Mixed Models (GLMMs) and Generalized Linear Models (GLMs) with a binomial distribution and logit link function, which are specifically designed for binary choice data (Side approach: Left vs. Right).

In Experiment 1, each fish was observed in a within-subjects design across four trials per Test and two Test conditions. To evaluate performance across all trials while accounting for the repeated-measures structure, we employed a GLMM (using the *lme4* package) with *Test Condition* as a fixed effect and *Fish ID* as a random intercept. To determine whether fish exhibited a systematic side bias, estimated marginal probabilities were compared against chance level (50%) using the emmeans package, with *p*-values adjusted for multiple comparisons using the Holm-Bonferroni correction. For first-trial data only, where each fish contributes a single binary choice, we used exact McNemar’s test (*exact2x2* package) to evaluate whether fish changed their choices between conditions more than expected by chance.

In Experiment 2, fish were assigned to different Training conditions in a between-subjects design, with each fish experiencing only one condition and being observed across four trials. For the analysis of all trials, we employed a GLMM with *Training Condition* as a fixed effect and *Fish ID* as a random intercept to account for multiple trials per individual. Comparisons against chance (50%) were performed using the same marginal probability estimation and Holm-adjusted post-hoc tests as in Experiment 1. For first-trial data, each fish’s binary choice was analyzed using a GLM (logistic regression). In this between-subjects design, direct group comparisons were performed using Type III Wald chi-square tests (for all trials) and Likelihood Ratio chi-square tests (for first trials).

Effect sizes were calculated using measures appropriate to each model type. For group comparisons (all trials and first trials), we calculated Odds Ratios (OR) with 95% confidence intervals. For post-hoc comparisons against chance, we calculated Cohen’s h using the formula h = 2[arcsin(sqrt(pobs))−arcsin(sqrt(0.5))], which uses an arcsine transformation to stabilize variance. For McNemar’s test, we calculated Cohen’s g = g = abs((b/(b+c))−0.5), where b and c represent the numbers of discordant pairs. All effect sizes were interpreted using conventional benchmarks for h (0.2 = small, 0.5 = medium, 0.8 = large) and g (0.05 = small, 0.15 = medium, 0.25 = large).

Confidence intervals (95%) were calculated to quantify estimation uncertainty. For all trials and group comparisons, CIs were derived directly from the model’s asymptotic standard errors on the logit scale and back-transformed to the response (probability) scale. For first-trial comparisons, CIs for the difference in proportions or odds were obtained using the profile likelihood method or normal approximation where appropriate. All tests were two-tailed.

Significant comparisons are reported in Figure 2 with asterisks ∗ indicating *p*-values < 0.05 (Holm-adjusted comparisons against chance level and between-condition comparisons).
