# Supplementary material for: Space-number association in zebrafish
Source: iScience. 2026 Nov 9;29(11):118179. doi: 10.1016/j.isci.2026.118179 (PMC13770723; doi:10.1016/j.isci.2026.118179)
Supplement: Supplementary file 1 [file mmc1.pdf]

iScience, Volume *29*

Supplemental information

Space-number association

in zebrafish

Davide Potrich, Mirko Zanon, Rosa Rugani, Valeria Anna Sovrano, and Giorgio Vallortigara

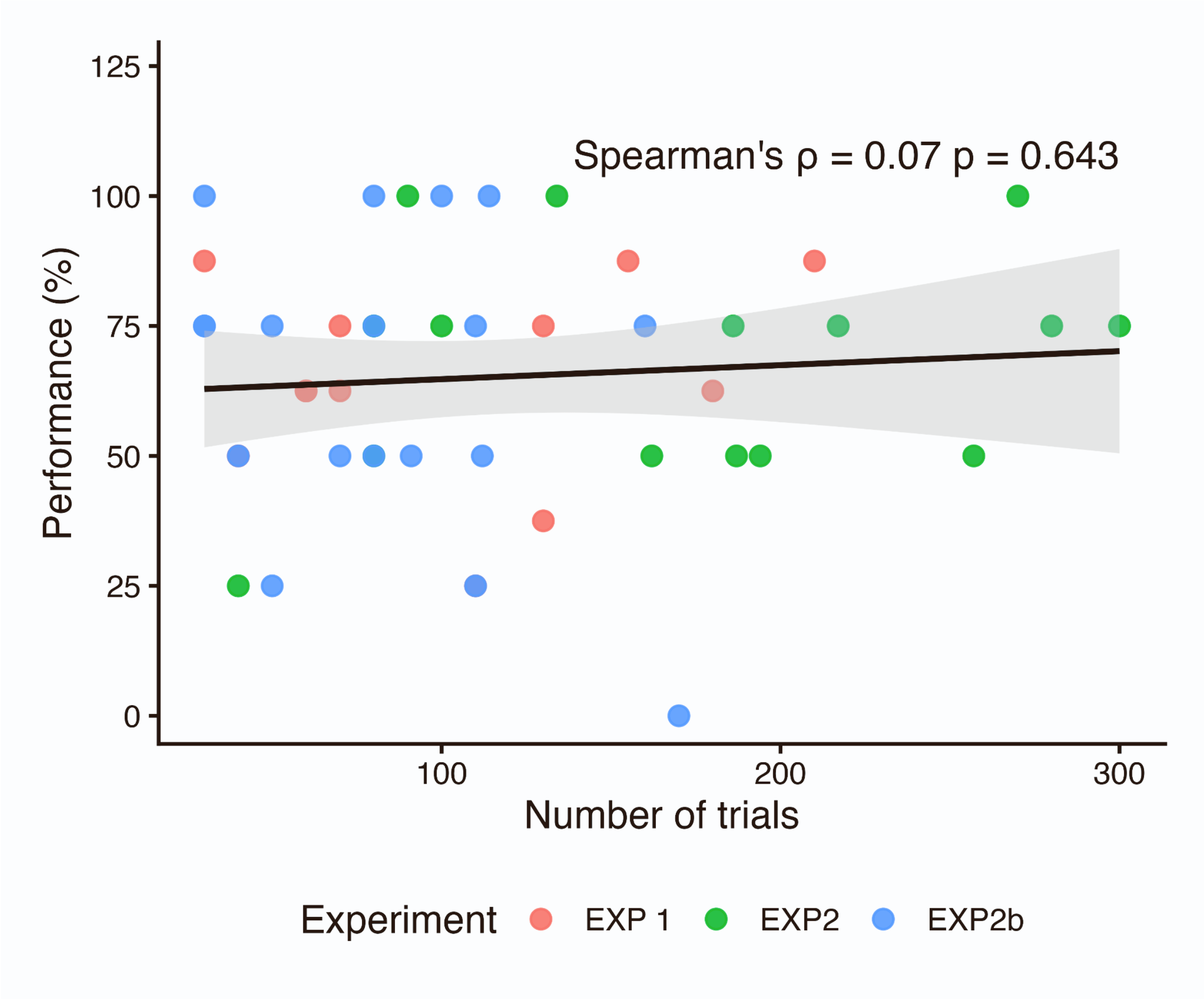

Figure S1. Correlation between performances and trials to criterion.
Relationship between the number of trials required to reach criterion and task performance across all experiments. Points represent individual fish and are coloured by experiment. The solid line shows the overall linear fit; Spearman's rank correlation (ρ) and associated *p*-value are reported.

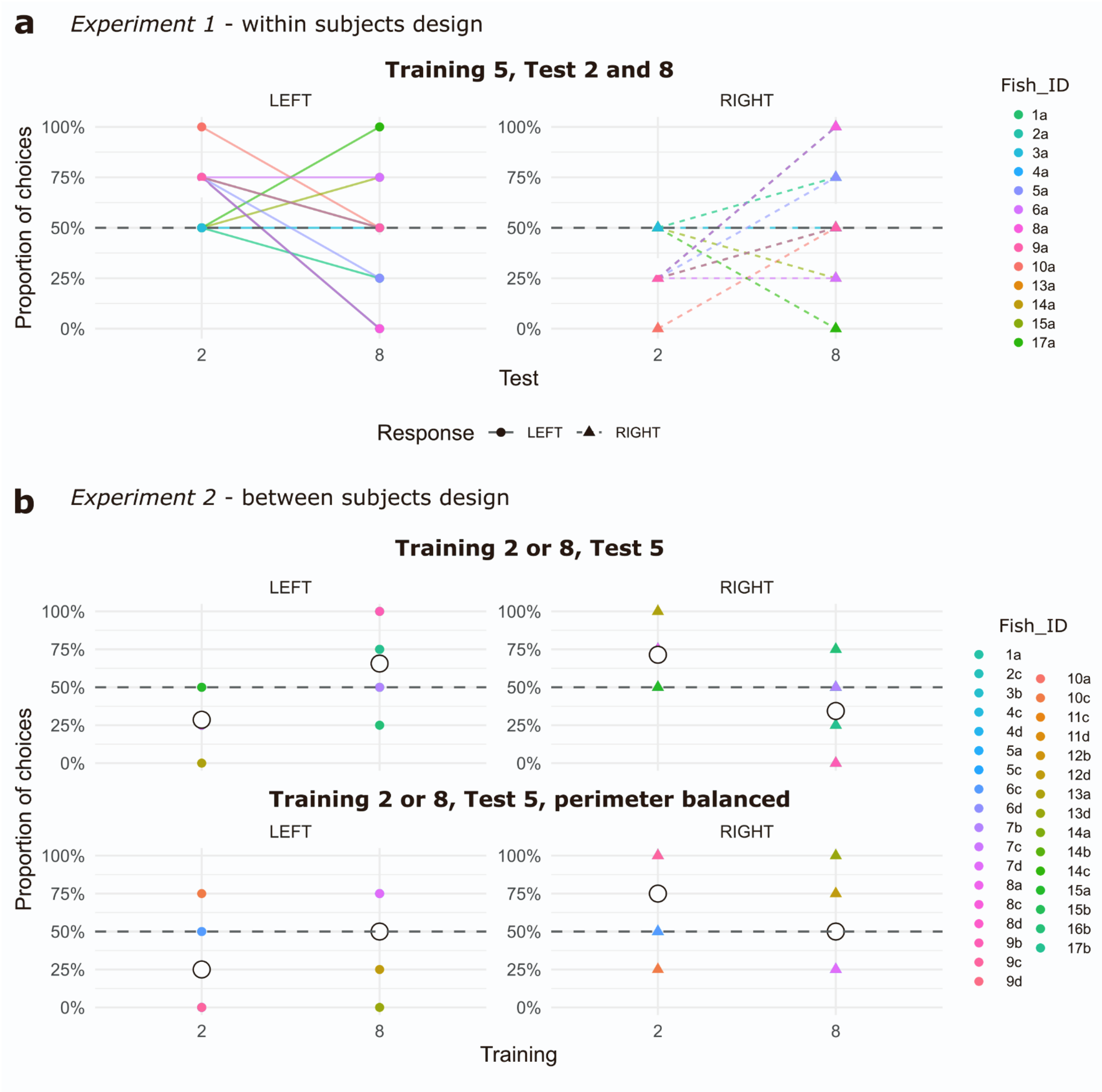

Figure S2. Proportion of choices per-fish.
Proportion of choices (over the 4 test trials) for the left and right side, for the different experiments. Each color represents a different fish. **a)** Data for Experiment 1 with Training numerosity 5 and Test 2+8 (within subjects); **b)** Data for Experiments 2 with Training numerosity 2 or 8 (between subjects) and Test 5 (both for no perimeter control -top- and perimeter controlled stimuli -bottom-). Note: between Experiment 1 and Experiment 2 different fish were used (the only fish reused for multiple tests were the ones in the within subjects design of Experiment 1).
